# Overexpression of HOTAIR leads to radioresistance of human cervical cancer via promoting HIF-1α expression

**DOI:** 10.1186/s13014-018-1153-4

**Published:** 2018-10-24

**Authors:** Ning Li, Dan-dan Meng, Ling Gao, Yue Xu, Pei-jie Liu, Yong-wei Tian, Zhen-ying Yi, Yan Zhang, Xiao-jing Tie, Zhi-qiao Xu

**Affiliations:** Tumor Diagnosis and Treatment Center of Kaifeng Central Hospital, No 85 HeDao street, Longting District, Kaifeng, 475001 Henan China

**Keywords:** Radiotherapy, Cervical cancer, *HOTAIR*, HIF-1α, Cell apoptosis

## Abstract

**Background:**

*HOTAIR* was known to enhance radioresistance in several cancers. However, the function of *HOTAIR* on radioresistance involving the regulation of HIF-1α in cervical cancer has not been reported.

**Methods:**

BALB/c nude mice were injected subcutaneously with HeLa cells and irradiated by X-ray. The tumor volume was measured and the expression of *HOTAIR* in tumors was detected by quantitative real-time PCR. Western blot was performed to detect the protein level of HIF-1α. MTT (3-(4,5-Dimethylthiazol-2-yl) 22,5-diphenyltetrazolium bromide) assay and the terminal deoxynucleotidyl transferase dUTP nick end labeling (TUNEL) assay was used to examine the cell viability and cell apoptosis of HeLa cells and C33A cells exposed to radiation.

**Results:**

Radiotherapy inhibited the tumor growth in mice bearing HeLa cells. Radiotherapy reduced the expression of *HOTAIR* and HIF-1α in tumor tissues and HeLa cells or C33A cells. *HOTAIR* overexpression abrogated the effect of radiation on the cell viability and cell apoptosis of HeLa and C33A cells. *HOTAIR* also upregulated the expression of HIF-1α in HeLa and C33A cell exposed to radiation. HIF-1α knockdown reversed increasing cell viability and reducing apoptosis of HeLa and C33A cell induced by *HOTAIR* overexpression. *HOTAIR* overexpression promoted tumor growth in mice bearing HeLa and exposed to radiation.

**Conclusion:**

Radiotherapy might inhibit cervical cancer cell growth through *HOTAIR*/HIF-1α pathway.

**Electronic supplementary material:**

The online version of this article (10.1186/s13014-018-1153-4) contains supplementary material, which is available to authorized users.

## Background

Cervical cancer is one of the most common gynecological malignancies worldwide, the morbidity and mortality of which increase year after year. To date, surgery, radiation therapy combined with chemotherapy is still mainly used for the treatment of patients with cervical cancer. Unfortunately, the effectiveness of radiotherapy and chemotherapy is poor and the five-year survival rate is only 40–50% [1]. Reports have demonstrated that hypoxia is an important factor impacting the effectiveness of radiotherapy and causing the tumor cells obtaining radioresistance [2]. Therefore, it is urgently required to elucidate the mechanisms underlying radioresistance of cervical cancer cells and improve the hypoxic condition in cells.

Owing to the rapid growth of tumor cells, vascular supplies are incapable of meeting the need of this growth, finally leading to hypoxia in tumor cells. The expression of several genes in hypoxic-tumor cells were altered, such as hypoxia-inducible factor 1 (HIF-1), causing the increase in cellular radioresistance [3]. Hypoxia inducible factor-1α (HIF-1α) is a nuclear factor existing widely in hypoxic tissues. HIF-1α is a key regulatory factor of cellular response to hypoxia, mediating the gene expression for cell survival and resistance to oxidative stress. It has been shown that HIF-1α regulated the expression of genes involving angiogenesis, tumor invasion, metastasis, proliferation and apoptosis [4]. Cui et al. reported that HIF-1α was highly expressed in cervical cancer tissues and associated with the decreasing radiosensitivity of cervical cancer [5]. However, the regulation of HIF-lα in cervical cancer is still poorly understood.

HOX transcript antisense intergenic RNA (*HOTAIR*) is an antisense transcript long noncoding RNA (lncRNA) located at the antisense strand of the HOXC gene locus in chromosome 12 q13.13 [6]. It has been shown that overexpression of *HOTAIR* contributed to the invasion and metastasis of several cancer cells, such as hepatocellular cancer, pancreatic carcinoma, breast cancer and colorectal cancer [7]. Higher level of *HOTAIR* was also found in colorectal cancer tissues comparing to that in the adjacent tissues, closely related to the patients’ age, clinical stages, invasive depth and lymphatic metastasis [8]. Studies have shown *HOTAIR* enhanced radioresistance in many cancers, such as breast cancer [9], colorectal cancer [10], pancreatic ductal adenocarcinoma [11], etc. However, the function of *HOTAIR* on the radioresistance of cervical cancer and the regulation of HIF-lα has not been reported.

In this study, we investigated the expression of *HOTAIR* in cervical cancer cells exposed to radiotherapy and analyzed the impact of *HOTAIR* and HIF-1α on the radiotherapy effect in cervical cancer cells.

## Methods

### Establishment of the nude mice model bearing cervical cancer cells

This experiment was performed in accordance with institutional guidelines of the Kaifeng Central Hospital and Use Committee guidelines. Six weeks old female BALB/c nude mice were randomly divided into two groups: control and radiation (*n* = 6 for each). At first, HeLa cells (1 × 10^6^ cells) were injected subcutaneously in the right posterior flank of the mice. After 10 days, mice were radiated with 10.8 Gy/each time for once a week, with totally four weeks (0, 7, 14 and 21 days, a total dose of 43.2 G) [13]. The large dose of radiation was aimed to determine the effect of radiation alone on tumor growth, which can obviously observe the effect of *HOTAIR* overexpression on radiation-inhibited tumor growth. The irradiation time lasted 30 min/each time. The X-ray was radiated by a Faxitron Cabinet X-ray System (Faxitron, IL, USA) (the dose rate = 0.36 Gy/min). Mice in control group did not receive radiation treatment. On day 26, all the mice were sacrificed and tumors were collected for measuring their volume (length×width^2^)/2 and detecting the expression of *HOTAIR* and HIF-1α.

### Cell culture and treatment

Human cervical cancer cell HeLa and C33A, normal cervical epithelial cells (NCECs) were obtained from the American Type Culture Collection (ATCC, VA, USA) and were cultured in RPMI1640 medium (Gibco, Gaithersburg, USA) with 10% fetal bovine serum (FBS; Gibco Gaithersburg, USA), and incubated at 37 °C in a humidifed incubator at 5% CO_2_. For the irradiation treatment, the exponentially growing cells were seeded in the culture flasks or culture dishes. When the cell confluence reached 60%, the cells were treated with 2 Gy for 0, 6, 12, and 24 h.

### Cell viability assay

3-(4,5-Dimethylthiazol-2-yl) 22,5-diphenyltetrazolium bromide (MTT) assay (ThermoFisher Scientific, CA, USA) was performed to detect the cell viability of cervical cancer cells as described by the manufacturer. HeLa cells or C33A cells were seeded into 96-well plates at an initial density of 3000 cells/well. After 48 h, the wells were incubated with MTT (5 mg/mL) for 4 h and the reaction was stopped by DMSO. Absorbance at 490 nm of the solution was read by using a spectrophotometric plate reader.

### Cell apoptosis analysis

The terminal deoxynucleotidyl transferase dUTP nick end labeling (TUNEL) assay was performed to detect the cell apoptosis using the In situ Apoptosis Detection Kit (Takara, Dalian, China). Cells were fixed by 4% paraformaldehyde/PBS solution for 30 min and washed by PBS for twice. After treated by 0.2% Triton X-100 for 5 min and washed by PBS for twice, cells were incubated with TdT reaction buffer and dUTP labelled by fluorescein for 1.5 h at 37 °C in humidifed chamber. Then washing buffer was added to stop the reaction. For viewing with a light microscope, the cells were incubated with converter-POD at 37 °C for 30 min in humidifed chamber. The reaction mixture was colored with DAB at room temperature for 10–15 min and terminated by washing with distilled water. The reaction mixture was counterstained with DAB for 20 min. TUNEL-positive nuclei were counted and detected with a light microscope (CX23, Olympus, Japan).

### Quantitative real-time PCR

The total RNA from cervical tumors or cervical cancer cells was collected by the Trizol reagent (Invitrogen) according to the manufacturer’s protocol. The purity and concentration of the total RNA was measured by using NanoDrop. The complementary DNA (cDNA) was synthesized using the First Strand cDNA Synthesis kit (Thermo Scientifc, USA) according to the manufacturers’ instructions. The expression of *HOTAIR* was quantified by real-time PCR using the SYBR EX TAQ (Takara, Dalian, China). GAPDH was used as the internal control. The relative expression of *HOTAIR* was calculated using the 2^−ΔΔCt^ method. Primers were used as follows: LncRNA HOTAIR: 5’-GTGGTGCTGACAAAGCTTGGAA-3′ (forward), 5’-TCACTGGGTGCCA TCGTAAGAA-3′(reverse);GAPDH: 5’-GGAGCGAGATCCCTCCAAAAT-3′ (forward), 5’-GGCTGTTGTCATACTTCTCATGG-3′ (reverse).

### Western blot

Total protein in cervical tumors or cervical cancer cells was extracted by using the RIPA buffer. Thirty micrograms of total protein were separated by 12% SDS-PAGE and then transferred onto the PVDF membrane (GE Health, London, UK). Following blocked in 5% non-fat milk for 1 h, the membrane was incubated with the primary antibodies overnight at 4 °C. The next day, the membrane was incubated with the horseradish peroxidaseconjugated second antibody for 2 h (Cell Signaling Technology, Boston, USA). Protein bands were visualized by using the ECL chemiluminescence kit (Pierce, IL, USA). The primary antibody for HIF-1α and β-Actin were purchased from Cell Signaling Technology (MN, USA).

### Cell transfection

The *HOTAIR* overexpressing (Ad-*HOTAIR*), HIF-1α overexpressing (Ad-HIF-1α), HIF-1α silencing (si-HIF-1α) or scrambled oligonucleotides ligated into the adenovirus vector was prepared by Riobio Co. Ltd. (Guangzhou, China). HeLa cells or C33A cells were seeded at 4 × 10^4^ cells/well in a 6-well plate, then transfected with Ad-*HOTAIR*, Ad-HIF-1α, si-HIF-1α or negative control adenovirus vector. The lenti-*HOTAIR* or scrambled oligonucleotides ligated into the lentiviral vector was prepared by Riobio Co. Ltd. (Guangzhou, China).HeLa cells transfected with Len-*HOTAIR* or Len-GFP were used to inject subcutaneously in the right posterior flank of the mice.

### Statistical analysis

All statistical analyses were performed using SPSS 17.0 (SPSS, Chicago, USA). All data were presented as mean ± standard deviations. Differences among groups were assessed by unpaired Student’s t test and one-way ANOVA. *P* < 0.05 was considered to be statistically significant.

## Results

### Radiotherapy inhibited the tumor growth in mice bearing HeLa and reduced the expression of *HOTAIR* and HIF-1α

**Fig. 1:**
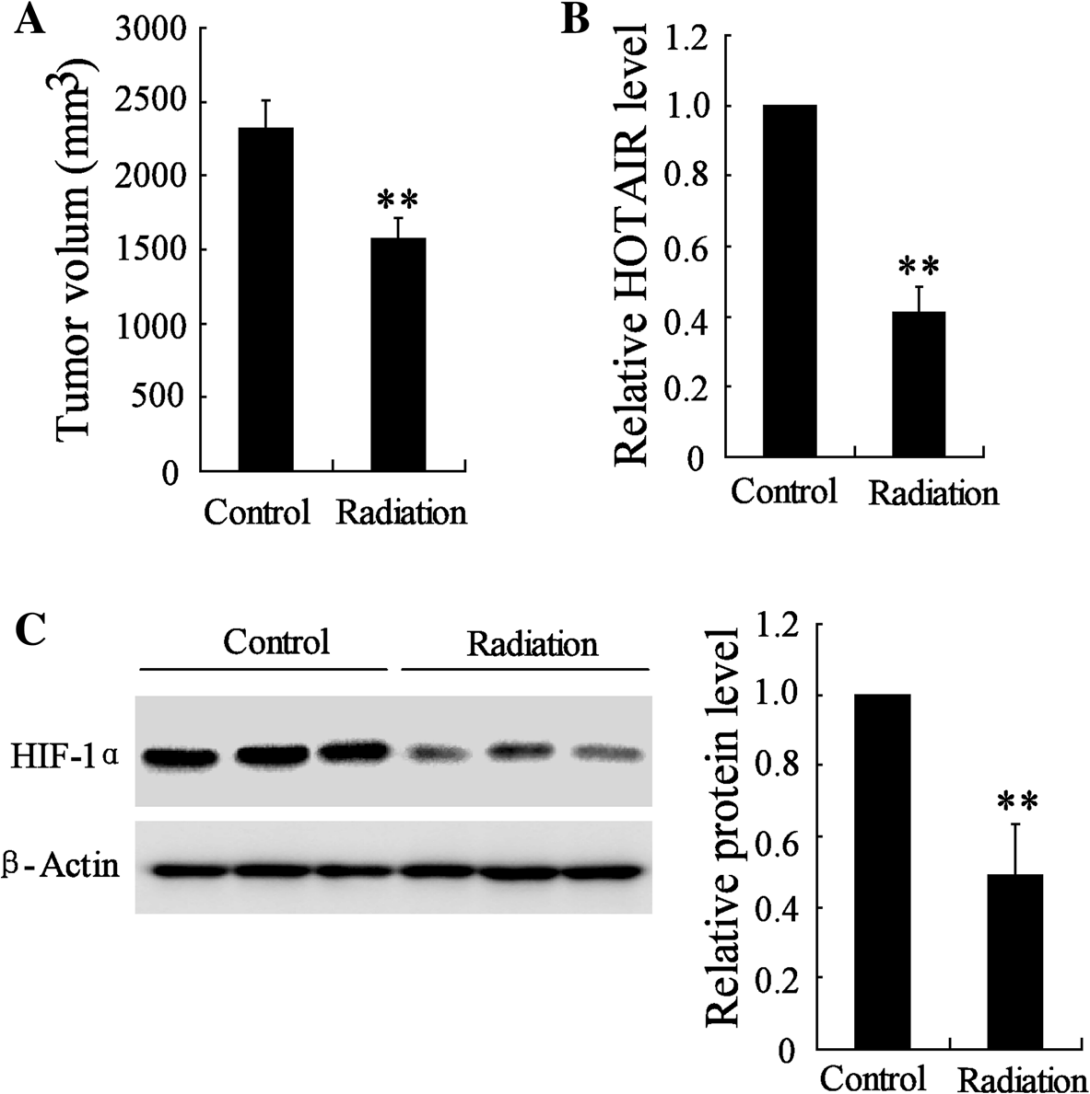
Effect of radiotherapy on the tumor growth in mice bearing HeLa cells and on the expression of *HOTAIR* and HIF-1α. Female BALB/c mice were divided into control group and radiation group. (**a**) The tumor growth in these mice after 4 weeks of radiotherapy. (**b**) The level of *HOTAIR* in tumor tissue of control group and radiation group. (**c**) The protein level of HIF-1α in tumor tissue of control group and radiation group. The data are represented as mean ± SD. *vs control, *P* < 0.01. All the experiment performed in triplicate

Compared with normal tissue, *HOTAIR* expression level increased 2.2 fold (*p* = 0.0045) in cervical cancer tissue, and HIF-1α expression was also upregulated in cervical cancer tissue (Additional file 1: Figure S1A). Female BALB/c mice were injected with cervical cancer cell HeLa and exposed to radiotherapy with 10.8 Gy/each time for once a week for a total of 4 weeks. The tumor growth decreased 0.68 fold by the radiation (*p* = 5.54 × 10^− 4^, Fig. 1a). The results of qRT-PCR indicated that the level of *HOTAIR* decreased 0.41 fold in cervical tumor of mice exposed to radiotherapy (*p* = 4.8 × 10^− 6^, Fig. 1b). We also detected the protein level of HIF-1α in cervical tumors of mice. Radiation suppressed the protein level of HIF-1α in cervical tumors (*p* = 3.64 × 10^− 4^, Fig. 1c).

### Radiation decreased the expression of *HOTAIR* and HIF-1α in HeLa cells and C33A cells

**Fig. 2:**
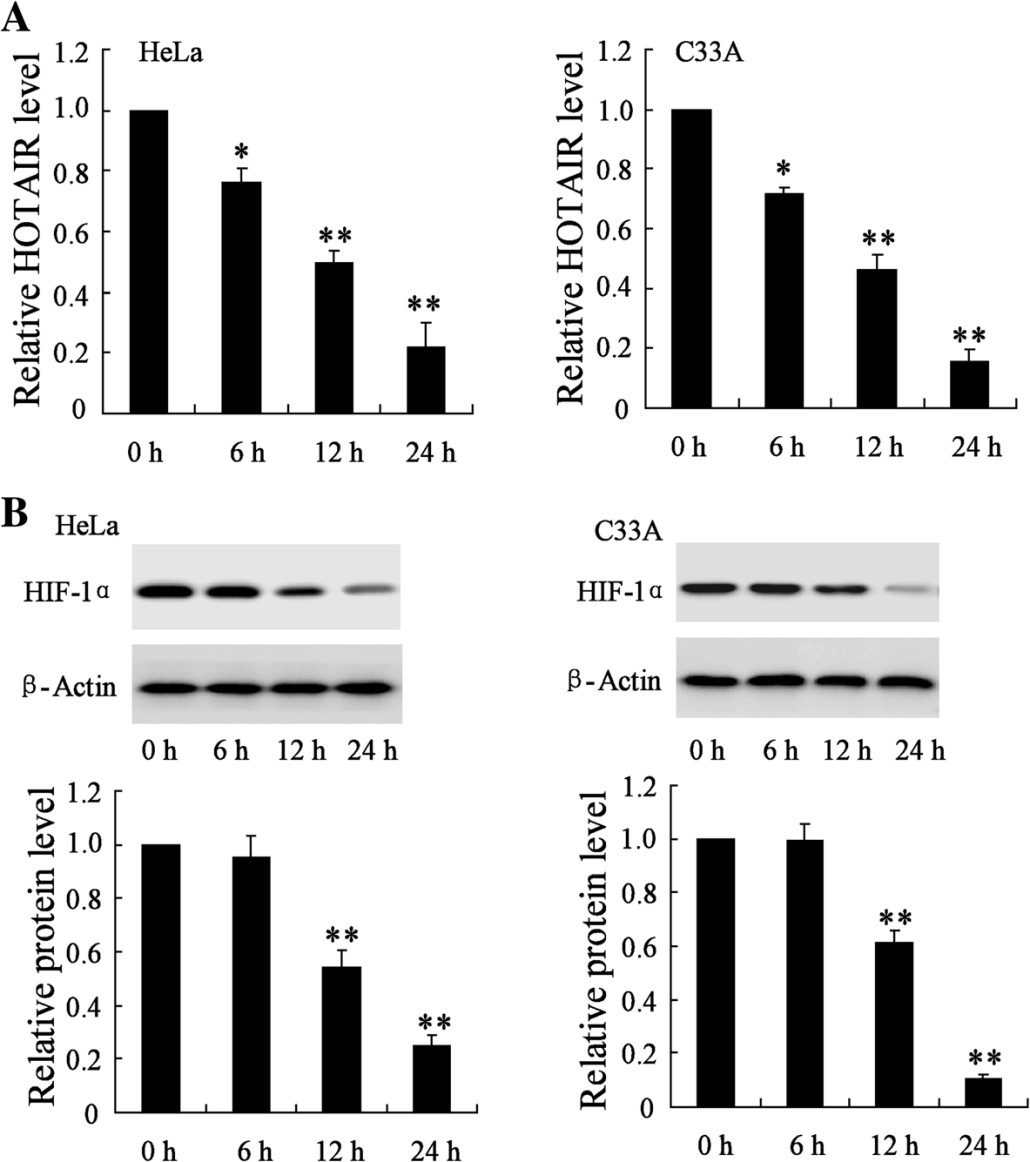
Effect of radiation on the expression of *HOTAIR* and HIF-1α in HeLa cells and C33A cells. HeLa cells and C33A cells were irradiated with a single dose of 2 Gy for 0 h, 6 h, 12 h, or 24 h. (**a**) The expression of *HOTAIR* in these HeLa cells and C33A cells. (**b**) The protein level of HIF-1α in these HeLa cells and C33A cells. The data are represented as mean ± SD. *vs control, *P* < 0.05; **vs control, *P* < 0.01. All the experiment performed in triplicate

Compared with NCECs, *HOTAIR* expression level was significantly upregulated in HeLa (2.87 fold, *p* = 2.95 × 10^− 5^) and C33A cells (2.33 fold, *p* = 3.76 × 10^− 4^), and HIF-1α expression was also upregulated in HeLa and C33A cells (Additional file 1: Figure S1B). The level of *HOTAIR* was significantly decreased in HeLa cells at 6 h, 12 h, 24 h after irradiation (0.76 fold, *p* = 5.97 × 10^− 4^; 0.50 fold, *p* = 3.07 × 10^− 5^; 0.15 fold, *p* = 8.3 × 10^− 5^) and C33A cells (0.71 fold, *p* = 3.89 × 10^− 5^; 0.46 fold, *p* = 5.46 × 10^− 5^; 0.16 fold, *p* = 3.5 × 10^− 6^) in a time-dependent manner. Particularly, the level of *HOTAIR* has declined by over 70% in HeLa cells and C33A cells 24 h after irradiation (Fig. 2a). As shown in Fig. 2b, the protein level of HIF-1α has no significant change in HeLa cells (0.95 fold, *p* = 0.34) and C33A cells (0.99 fold, *p* = 0.80) 6 h after irradiation comparing to the control. While, with the exposure time longer, the protein level of HIF-1α was significantly downregulated in HeLa cells at 12 h and 24 h after irradiation (0.54 fold, *p* = 2.63 × 10^− 4^; 0.25 fold, *p* = 5.36 × 10^− 6^) and C33A cells (0.61 fold, *p* = 1.2 × 10^− 4^; 0.11 fold, *p* = 5.7 × 10^− 8^) (Fig. 2b).

### *HOTAIR* overexpression abrogated the effect of radiation on the cell viability and cell apoptosis of HeLa and C33A cell

**Fig. 3:**
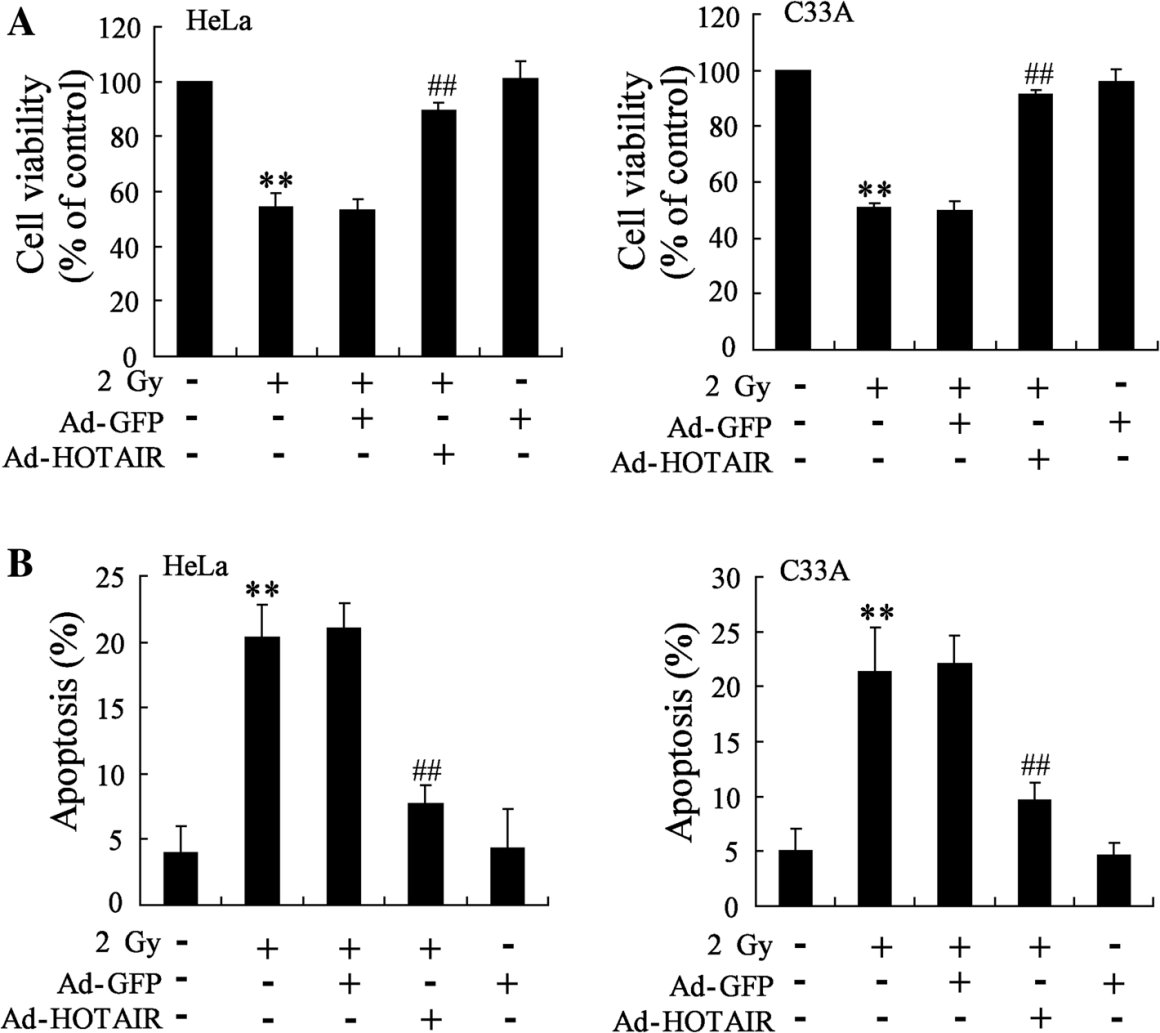
Effect of *HOTAIR* overexpression on cell viability and apoptosis of HeLa cells and C33A cells exposed to radiation. HeLa and C33A cells were divided into control, radiation, radiation+Ad-GFP, radiation+Ad-*HOTAIR* groups. (**a**) MTT assay was performed to detect the cell viability of HeLa and C33A cells. (**b**) Cell apoptosis of HeLa and C33A cells was examined by TUNEL assay. The data are represented as mean ± SD. **vs control cells, *P* < 0.01; ##vs cells irradiated with a dose of 2 Gy and transfected with Ad-GFP, *P* < 0.01. All the experiment performed in triplicate

As we have known that *HOTAIR* might be associated with the effect of radiation on tumor, the impact of *HOTAIR* overexpression on cell viability and cell apoptosis was detected in cells exposed to radiation. Our results showed that radiation indeed inhibited the cell viability and increased the cell apoptosis of HeLa (5.08 fold, *p* = 9.2 × 10^− 4^) and C33A cells (4.27 fold, *p* = 0.003) comparing to control (Fig. 3). In addition, *HOTAIR* overexpression abrogated the effect of radiation on the cell viability and cell apoptosis of HeLa (0.38 fold, *p* = 7.8 × 10^− 4^) and C33A cells (0.45 fold, *p* = 0.002) (Fig. 3).

### *HOTAIR* overexpression upregulated the expression of HIF-1α in HeLa and C33A cell exposed to radiation

**Fig. 4:**
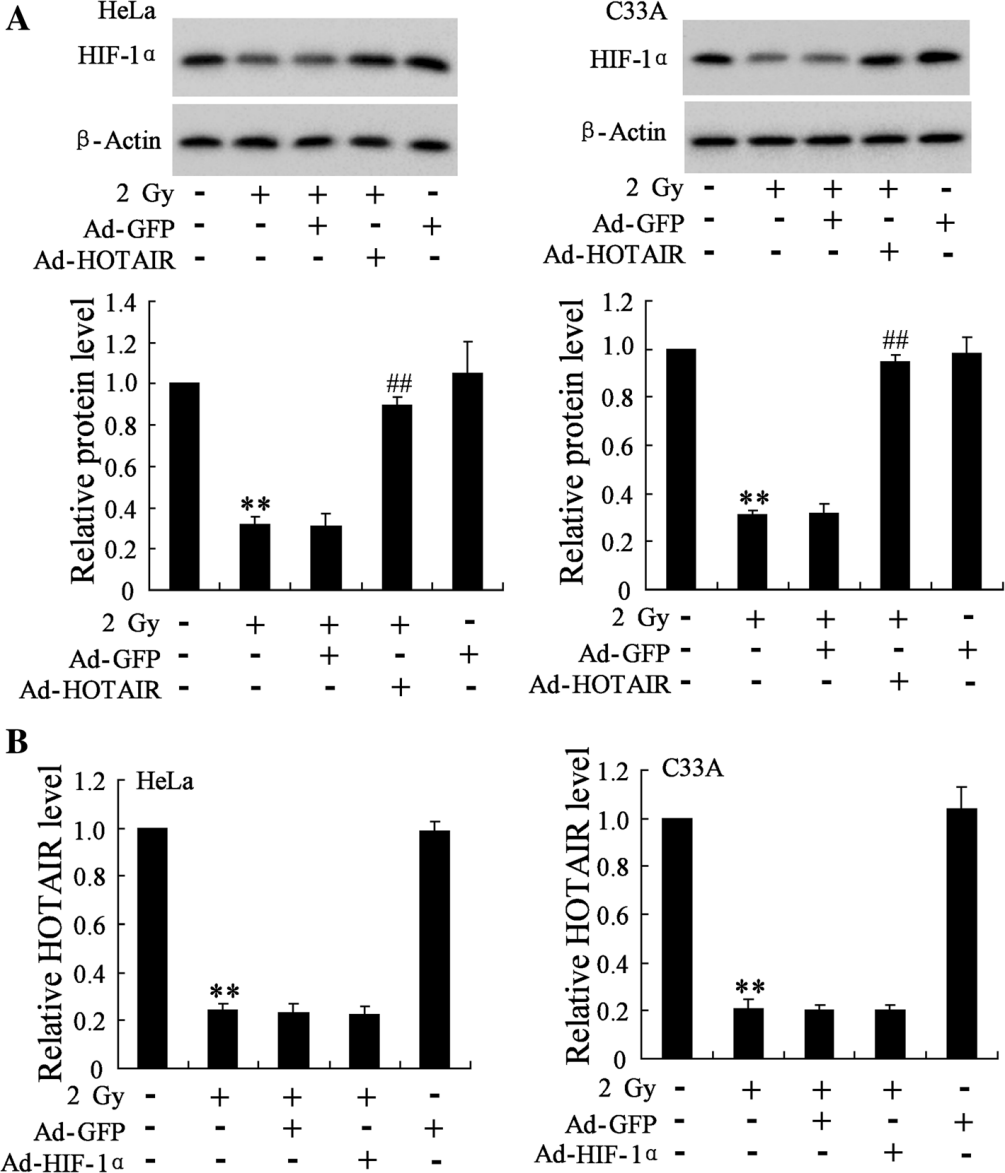
Effect of *HOTAIR* overexpression on HIF-1α expression in HeLa and C33A cells exposed to radiation. (**a**) The protein level of HIF-1α in HeLa and C33A cells was detected. (**b**) The expression of *HOTAIR* in HeLa and C33A cells was detected. The data are represented as mean ± SD. **vs control cells, *P* < 0.01; ##vs cells irradiated with a dose of 2 Gy and transfected with Ad-GFP, *P* < 0.01. All the experiment performed in triplicate

*HOTAIR* overexpression significantly increased the protein level of HIF-1α in HeLa and C33A cells exposed to radiation and transfected with Ad-*HOTAIR* (2.8 fold, *p* = 1.82 × 10^− 4^) comparing to cells exposed to radiation and transfected with Ad-GFP (2.9 fold, *p* = 9.11 × 10^− 6^), which could be abolished by miR-217 mimic (Fig. 4a, Additional file 2: Figure S2). Therefore, we speculated that in cervical cancer cells, hypoxia upregulated HOTAIR expression to sponge miR-217, thus to promote HIF-1α expression. As HIF-1α was reported to upregulate the expression of *HOTAIR* at transcriptional level in hypoxic cancer cells, we examined the effect of HIF-1α overexpression on the level of *HOTAIR* in HeLa and C33A cells. HIF-1α overexpression could not reverse the downregulation of *HOTAIR* induced by radiation in HeLa (0.92 fold, *p* = 0.77) and C33A cells (0.95 fold, *p* = 0.87) (Fig. 4b, Additional file 2: Figure S2).

### HIF-1α knockdown reversed increasing cell viability and reducing apoptosis of HeLa and C33A cell induced by *HOTAIR* overexpression

**Fig. 5:**
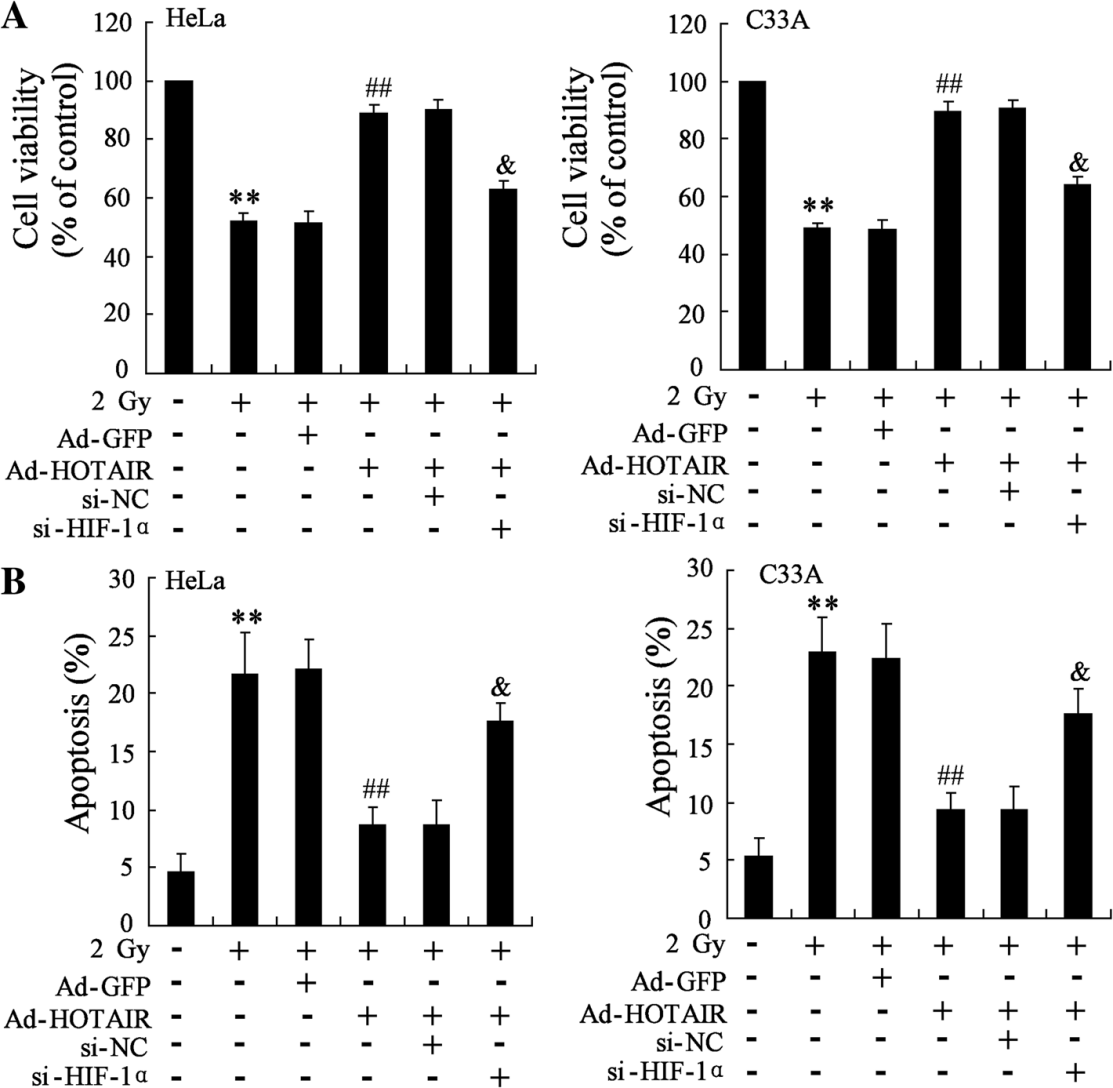
Effect of HIF-1α knockdown on the cell viability and apoptosis of HeLa and C33A cell exposed to radiation. HeLa and C33A cells were divided into six groups: control, radiation, radiation+Ad-GFP, radiation+Ad-*HOTAIR*, radiation+Ad-*HOTAIR* + si-NC, and radiation+Ad-*HOTAIR* + si-HIF-1α groups. The cell viability (**a**) and apoptosis (**b**) of HeLa and C33A cell exposed to radiation were examined. The data are represented as mean ± SD. **vs control cells, *P* < 0.01; ##vs cells irradiated with a dose of 2 Gy and transfected with Ad-GFP, *P* < 0.01; &vs cells irradiated with a dose of 2 Gy and transfected with Ad-*HOTAIR* and si-NC (scrambled sequence), *P* < 0.01. All the experiment performed in triplicate

To investigate the effect of HIF-1α knockdown on the cell viability and apoptosis of cells exposed to radiation, HeLa and C33A cells were subjected to radiation with 2 Gy for 24 h and transfected with Ad-*HOTAIR* and (or) si-HIF-1α. HIF-1α knockdown reversed the increasing cell viability of HeLa (0.72 fold, *p* = 5.68 × 10^− 4^) and C33A cells (0.72 fold, *p* = 3.34 × 10^− 4^) and decreasing apoptosis of HeLa (2.03 fold, *p* = 0.003) and C33A cells (1.89 fold, *p* = 0.008) induced by *HOTAIR* overexpression (Fig. 5).

### *HOTAIR* overexpression promoted tumor growth in mice bearing HeLa and exposed to radiation

**Fig. 6:**
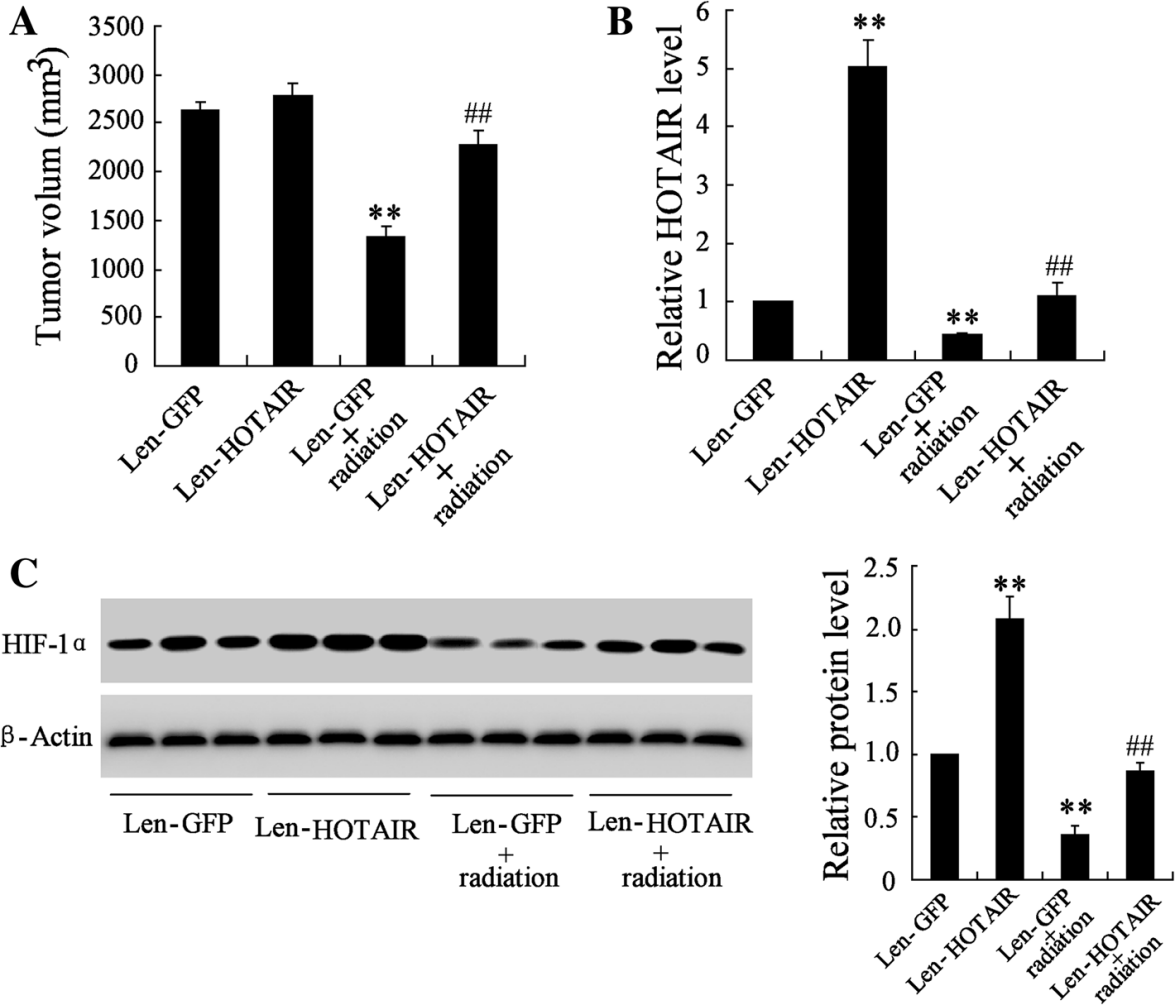
Effect of *HOTAIR* overexpression on tumor growth in mice bearing HeLa cells and exposed to radiation. Mice bearing HeLa cells were divided into four groups: Len-GFP, Len-*HOTAIR*, Len-GFP+ radiation and Len-*HOTAIR*+ radiation. (**a**) The tumor growth was measured in these mice. (**b**) The expression of *HOTAIR* in these mice. (**c**) The protein level of HIF-1α in these mice. **vs Len-GFP, *P* < 0.01; ##vs Len-GFP + radiation, *P* < 0.01. All the experiment performed in triplicate

As shown in Fig. 6a, Len-*HOTAIR* significantly promoted the tumor growth in Len-HOTAIR+radiation group comparing to Len-GFP + radiation group (1.71 fold, *p* = 3.07 × 10^− 5^). We found that Len-*HOTAIR* significantly increased the level of *HOTAIR* (5.02 fold, *p* = 2.16 × 10^− 6^) and the protein level of HIF-1α (2.08 fold, *p* = 2.43 × 10^− 5^), and radiation significantly decreased *HOTAIR* (0.44 fold, *p* = 7.58 × 10^− 4^) and HIF-1α expression (0.36 fold, *p* = 7.05 × 10^− 5^) (Fig. 6b and c). Mice bearing C33A cells transfected with Len-*HOTAIR* had similar results, shown in Additional file 3: Figure S3.

## Discussion

To date, radiotherapy has been one of the major therapies for cancer treatment. Radiotherapy technology for cervical cancer is also increasingly innovated with the application of three dimensional conformal radiation therapy and intensity modulated radiationtherapy [14, 15]. However, the radiosensitivity developed in tumor cells greatly compromises the effectiveness of radiotherapy. Long noncoding RNAs (lncRNAs) have been known to play a crucial role in the regulation of cellular processes, as well as the cancer radiation resistance [16]. Among the extensively studied lncRNAs, *HOTAIR* was found to be associated with the mechanism of radiosensitivity in several cancers [17]. For example, *HOTAIR* affected the radiosensitivity of pancreatic ductal adenocarcinoma by regulating the expression of Wnt inhibitory factor 1 [18]. Chen reported that radiotherapy induced Lewis lung cancer cell apoptosis via inactivating β-catenin mediated by upregulating *HOTAIR* [12].

In the present study, we found that *HOTAIR* was greatly decreased in cervical tumor from mice and cervical cancer cells exposed to radiotherapy. In addition, *HOTAIR* overexpression abrogated the effect of radiation on the cell viability and cell apoptosis of HeLa and C33A cells. *HOTAIR* overexpression slightly promoted the tumor growth in mice injected with Len-*HOTAIR* comparing to that in mice injected with Len-GFP. Furthermore, Len-*HOTAIR* abrogated the inhibition effect of radiation on tumor growth in mice. Li et al. reported that *HOTAIR* induced radio-resistance and enhanced aggressive biological behaviors via inhibiting p21 in cervical cancer, which indicated that *HOTAIR* might be a potent therapeutic strategy for those cervical cancer patients especially who accepted radiotherapy [17]. Evidences have showed the significant diagnostic and prognostic value of *HOTAIR* for cervical cancer [8, 19].

Hypoxia is an important factor contributing to the radioresistance of tumor cells. Meanwhile, accumulating studies have revealed that depletion of oxygen leads to the inefficient of radiation on DNA strand breaks [20]. Hypoxic stimuli might increase the cellular radioresistant phenotype through changing the cell death/survival signaling pathway and DNA damage repair pathway [21]. In hypoxia-related tumor radioresistance, HIF-1α plays significant roles and is an important prognostic factor after radiation therapy [22].

To investigate the mechanism of *HOTAIR*-induced radio-resistance involving hypoxia, we explored the expression of HIF-1α and its regulation. Radiation decreased the expression of HIF-1α in cervical tumor from mice and cervical cancer cells. *HOTAIR* overexpression significantly increased the protein level of HIF-1α in HeLa and C33A cells exposed to radiation. As HIF-1α was reported to upregulate the expression of *HOTAIR* at transcriptional level in hypoxic cancer cells, we examined the effect of HIF-1α overexpression on the level of *HOTAIR* in HeLa and C33A cells. However, HIF-1α overexpression could not reverse the downregulation of *HOTAIR* induced by radiation in HeLa and C33A cells. We also found that HIF-1α knockdown reversed increasing cell viability and reducing apoptosis of HeLa and C33A cell induced by *HOTAIR* overexpression. Bachtiary et al. reported that overexpression of HIF-1α had predictive and prognostic significance in cervical cancer patients receiving curative radiation therapy [23]. HIF-1α also protected cervical carcinoma cells from apoptosis induced by radiation via modulation of vascular endothelial growth factor and p53 under hypoxia [24]. In the current study, we found that *HOTAIR* overexpression induced the radioresistance through upregulating the expression of HIF-1α in cervical cancer cells. However, the mechanism of how *HOTAIR* interacted with HIF-1α in cervical cancer cells exposed to radiotherapy needs to be further investigated.

## Conclusions

In conclusion, the present study not only reveals that radiotherapy inhibits the cervical cancer cell growth through downregulating *HOTAIR* to inhibit the expression of HIF-1α, but also sheds new lights on the molecular mechanisms related to radioresistance of cervical cancer cell. Furthermore, it implicates that *HOTAIR*-HIF-1α axis might be a potential target for cervical cancer radiotherapy.

## Additional files

Additional file 1: **Figure S1**. *HOTAIR* and HIF-1α expression in cervical cancer tissues and cells. A. *HOTAIR* and HIF-1α expression was upregulated in cervical cancer tissues than normal tissues. B. *HOTAIR* and HIF-1α expression was upregulated in cervical cancer cells than normal cervical epithelial cells (NCECs). **vs normal tissue or NCECs, *p* < 0.01. (TIF 588 kb)

Additional file 2: **Figure S2**. Effect of *HOTAIR* overexpressing on HIF-1α expression in HeLa and C33A cells. **vs 0 Gy, *P* < 0.01; ##vs 2 Gy + Ad-GFP, P < 0.01; &vs 2 Gy + Ad-HOTAIR+Pre-NC, *P* < 0.01. (TIF 707 kb)

Additional file 3: **Figure S3**. Effect of *HOTAIR* overexpression on tumor growth in mice bearing C33A cells and exposed to radiation. Mice bearing C33A cells were divided into four groups: Len-GFP, Len-*HOTAIR*, Len-GFP + radiation and Len-*HOTAIR* + radiation. A. The tumor growth was measured in these mice. (B) The expression of *HOTAIR* in these mice. (C) The protein level of HIF-1α in these mice. **vs Len-GFP, *P* < 0.01; ##vs Len-GFP + radiation, *P* < 0.01. (TIF 766 kb)

## Abbreviations

cDNA: complementary DNA
HIF-1: hypoxia-inducible factor 1
HIF-1α: Hypoxia inducible factor-1α
*HOTAIR*: HOX transcript antisense intergenic RNA
lncRNA: noncoding RNA
TUNEL: transferase dUTP nick end labeling
