# Supplementary figures and images for: Overexpression of HOTAIR leads to radioresistance of human cervical cancer via promoting HIF-1α expression
Source: Radiat Oncol. 2018 Oct 24;13:210. doi: 10.1186/s13014-018-1153-4 (PMC6201557; doi:10.1186/s13014-018-1153-4)

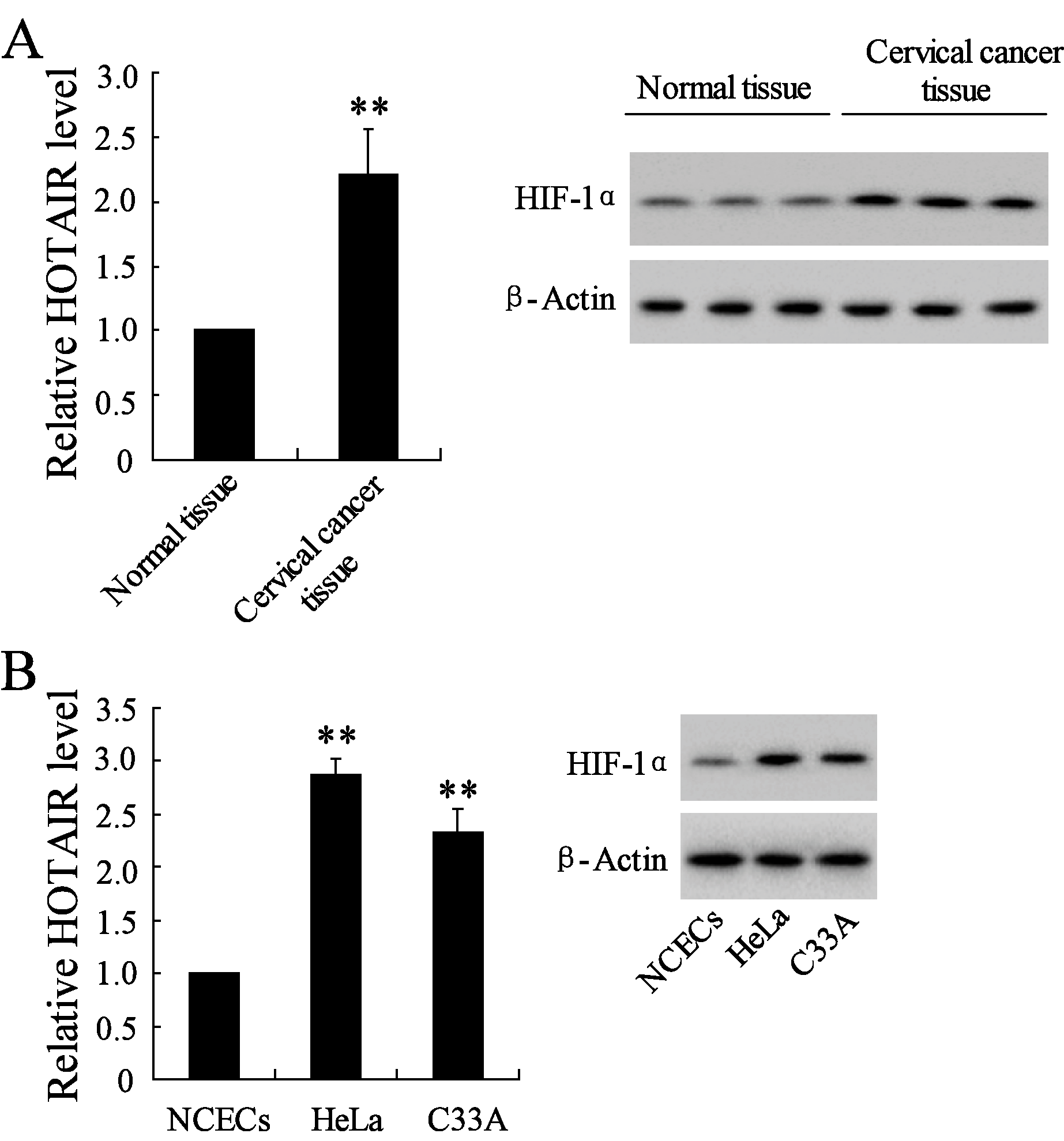

Supplement: Supplementary file 1 — Figure S1. HOTAIR and HIF-1α expression in cervical cancer tissues and cells. A. HOTAIR and HIF-1α expression was upregulated in cervical cancer tissues than normal tissues. B. HOTAIR and HIF-1α expression was upregulated in cervical cancer cells than normal cervical epithelial cells (NCECs). **vs normal tissue or NCECs, p < 0.01. (TIF 588 kb) [file 13014_2018_1153_MOESM1_ESM.tif]

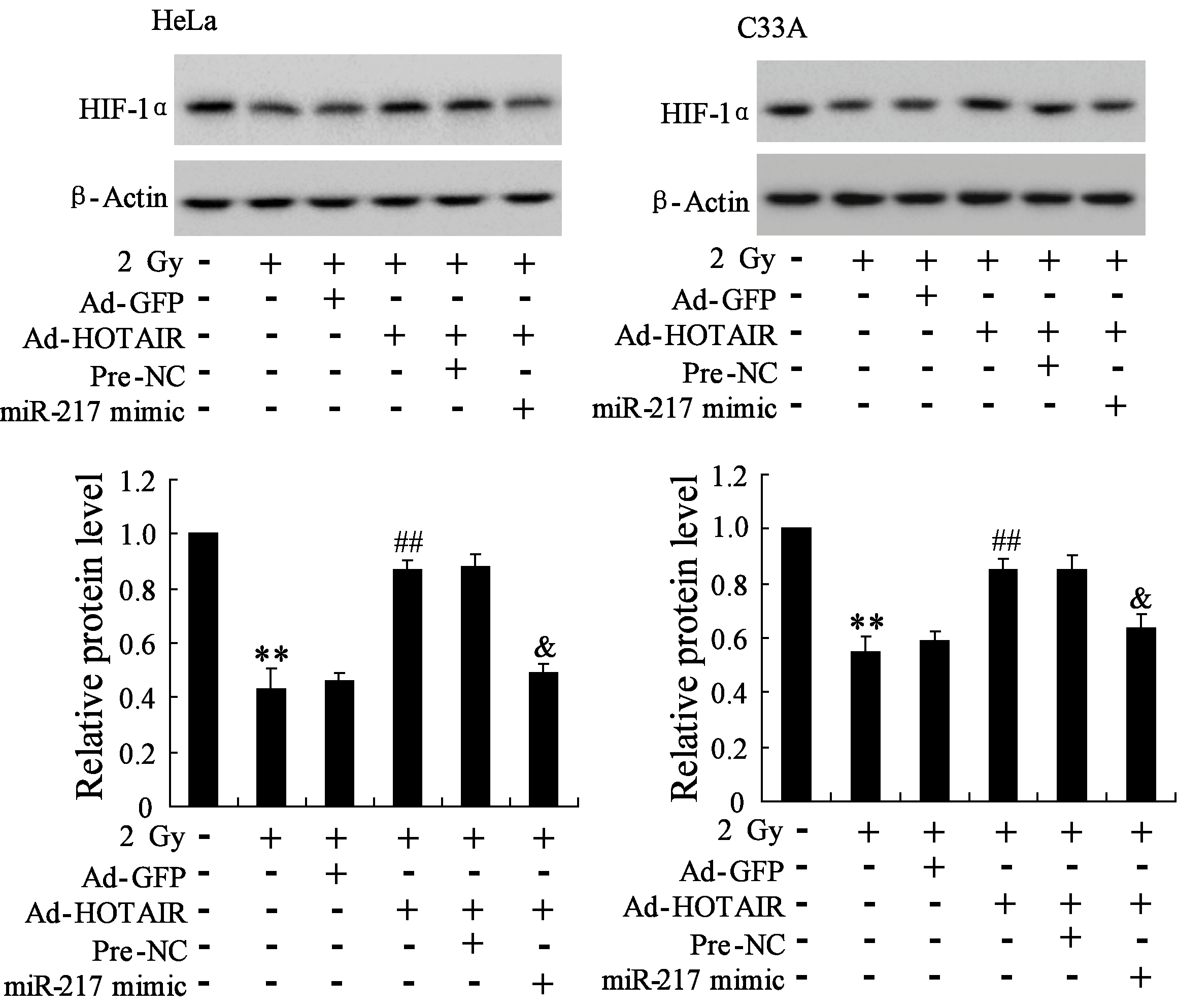

Supplement: Supplementary file 2 — Figure S2. Effect of HOTAIR overexpressing on HIF-1α expression in HeLa and C33A cells. **vs 0 Gy, P < 0.01; ##vs 2 Gy + Ad-GFP, P < 0.01; &vs 2 Gy + Ad-HOTAIR+Pre-NC, P < 0.01. (TIF 707 kb) [file 13014_2018_1153_MOESM2_ESM.tif]

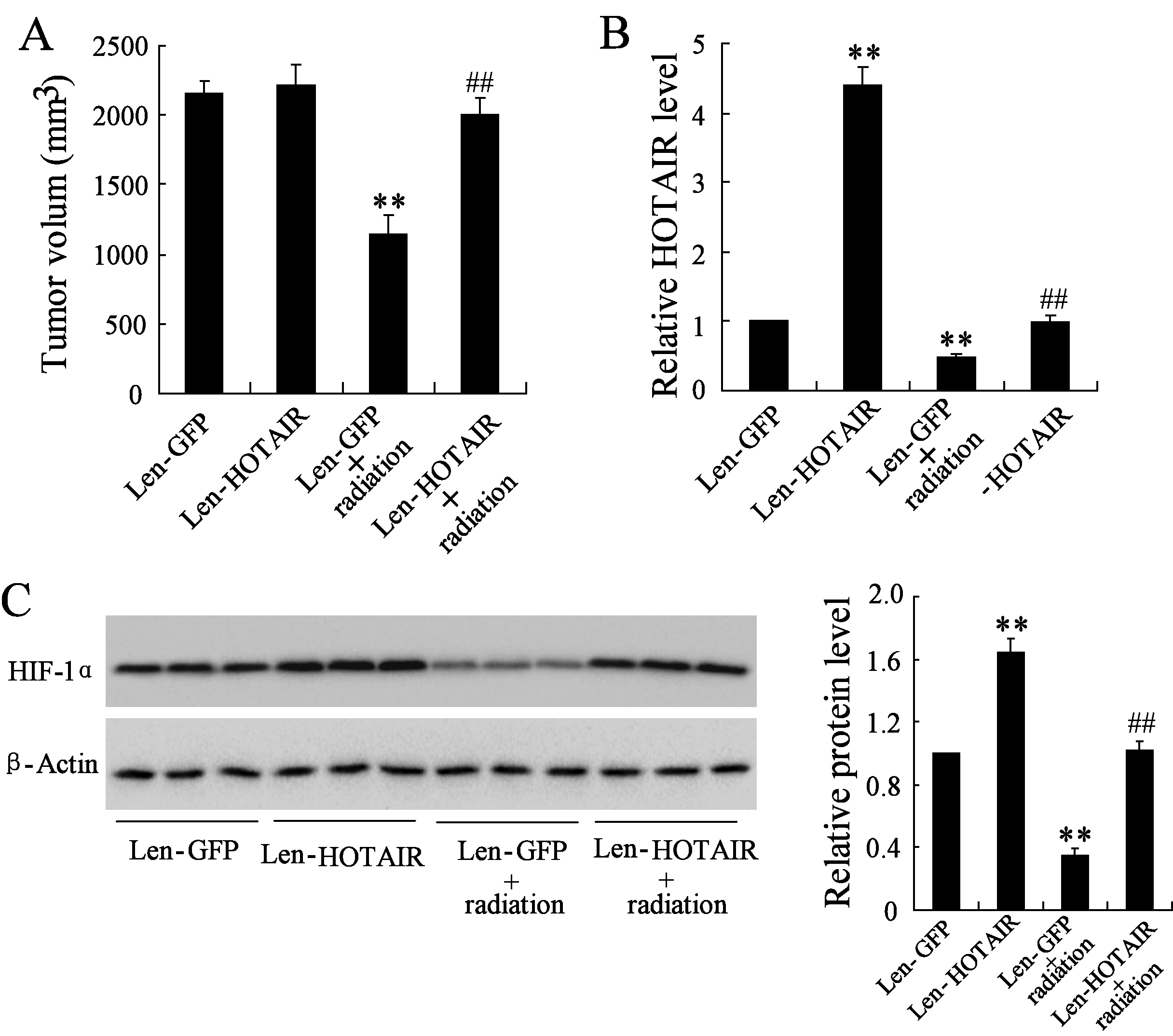

Supplement: Supplementary file 3 — Figure S3. Effect of HOTAIR overexpression on tumor growth in mice bearing C33A cells and exposed to radiation. Mice bearing C33A cells were divided into four groups: Len-GFP, Len-HOTAIR, Len-GFP + radiation and Len-HOTAIR + radiation. A. The tumor growth was measured in these mice. (B) The expression of HOTAIR in these mice. (C) The protein level of HIF-1α in these mice. **vs Len-GFP, P < 0.01; ##vs Len-GFP + radiation, P < 0.01. (TIF 766 kb) [file 13014_2018_1153_MOESM3_ESM.tif]
